# Seroprevalence and risk factors associated with bovine brucellosis in the Potohar Plateau, Pakistan

**DOI:** 10.1186/s13104-017-2394-2

**Published:** 2017-01-28

**Authors:** Shahzad Ali, Shamim Akhter, Heinrich Neubauer, Falk Melzer, Iahtasham Khan, Emmanuel Nji Abatih, Hosny El-Adawy, Muhammad Irfan, Ali Muhammad, Muhammad Waqas Akbar, Sajid Umar, Qurban Ali, Muhammad Naeem Iqbal, Abid Mahmood, Haroon Ahmed

**Affiliations:** 1 University of Veterinary and Animal Sciences, Lahore, Pakistan; 2 Pir Mehr Ali Shah Arid Agriculture University Rawalpindi, Rawalpindi, Pakistan; 3 Friedrich-Loeffler-Institut, Institute of Bacterial Infections and Zoonoses, Naumburger Str. 10 96a, 07743 Jena, Germany; 4 Unit of Epidemiology and Biostatistics, Department of Biomedical Sciences, Institute of Tropical Medicine, Antwerp, Belgium; 5 Faculty Medicineof Veterinary, Kafrelsheikh University, Kafr El-Sheikh, Egypt; 6 National Veterinary Laboratories, Islamabad, Pakistan; 7 Department of Biosciences, COMSATS Institute of Information Technology, Park Road,Chak Shahzad, Islamabad, Pakistan; 8 Department of Wildlife and Ecology (Zoological Division), University of Veterinary and Animal Sciences, Lahore, Pakistan

**Keywords:** Bovine brucellosis, Serology, Bacteriology, qRT-PCR, Risk factors, Pakistan

## Abstract

**Background:**

The seroprevalence and risk factors of bovine brucellosis were studied at animal and herd level using a combination of culture, serological and molecular methods. The study was conducted in 253 randomly selected cattle herds of the Potohar plateau, Pakistan from which a total of 2709 serum (1462 cattle and 1247 buffaloes) and 2330 milk (1168 cattle and 1162 buffaloes) samples were collected. Data on risk factors associated with seroprevalence of brucellosis were collected through interviews using questionnaires. Univariable and multivariable random effects logistic regression models were used for identifying important risk factors at animal and herd levels.

**Results:**

One hundred and seventy (6.3%) samples and 47 (18.6%) herds were seropositive for brucellosis by Rose Bengal Plate test. Variations in seroprevalence were observed across the different sampling sites. At animal level, sex, species and stock replacement were found to be potential risk factors for brucellosis. At herd level, herd size (≥9 animals) and insemination method used were important risk factors. The presence of *Brucella* DNA was confirmed with a real-time polymerase chain reaction assay (qRT-PCR) in 52.4% out of 170 serological positive samples. In total, 156 (6.7%) milk samples were positive by milk ring test. *B. abortus* biovar 1 was cultured from 5 positive milk samples.

**Conclusion:**

This study shows that the seroprevalence of bovine brucellosis is high in some regions in Pakistan. Prevalence was associated with herd size, abortion history, insemination methods used, age, sex and stock replacement methods. The infected animal may act as source of infection for other animals and for humans. The development of control strategies for bovine brucellosis through implementation of continuous surveillance and education programs in Pakistan is warranted.

**Electronic supplementary material:**

The online version of this article (doi:10.1186/s13104-017-2394-2) contains supplementary material, which is available to authorized users.

## Background

Brucellosis remains an important zoonotic disease in animals and humans. It is mainly caused by *B. abortus* (cattle and buffaloes), *B. melitensis* (sheep and goats), and *B. suis* (pigs) [1]. This disease has a considerable negative impact on socioeconomic aspects in Mediterranean countries, countries of Central Asia and especially in the rural areas of developing countries, where livestock rearing and production of dairy products and by-products is crucial for family income [2]. In humans, the disease spreads through the infected food-chain via milk and dairy products [3, 4]. Brucellosis is considered as an occupational hazard with humans particularly at risk either living in close proximity with infected animals, handling them or even consume their products. It is a public health problem in developing countries like Pakistan with adverse health implications for animals and human beings and economic implications for individuals and communities [3].

In bovines, *B. abortus* is the most frequent causative agent. Apart from *B. abortus*, occasionally *B. melitensis* and *B. suis* cause brucellosis in bovines if kept together with sheep and goats or pigs, respectively [5, 6]. *B. abortus* has been eradicated from Japan, Canada, various northern and central European countries, Australia, New Zealand and from farmed cattle in the U.S.A. [7]. Abortion is the most common sign of disease in bovines. Other clinical signs include infertility, repeated insemination, reduction of milk production, retention of the placenta, metritis, arthritis, epididymitis and orchitis [2, 8]. Risk factors associated with animal/herd level brucellosis like herd size, husbandry system, veterinary extension services, use of disinfectants and abortion rate have been studied in different regions by various authors [9, 10].

Livestock is the major source of income for 30–40% of people in the rural areas of Pakistan, where 30–35 million persons are engaged in raising livestock. The dairy sector in Pakistan plays a pivotal role in the national economy and its value is more than that of the wheat and cotton sectors combined. Estimated annual milk production in 2014/2015 was approximately 52.6 million l, ranking Pakistan one of the world’s top milk producers [11]. Animals in Pakistan are affected by many diseases, among them brucellosis in bovines caused by *B. abortus* biovar 1 [12, 13]. Prevalence of bovine brucellosis (3–6.5%) based on serological tests has been reported from different areas of Pakistan [14, 15]. Previous studies showed a seroprevalence of 6.9% and 30.5% in humans coming from two different areas of Pakistan [9, 16]. Recently, a seroprevalence was reported in cattle using Rose Bengal plate test (RBPT) (10.2%) and enzyme-linked immunosorbent assay (ELISA) (8%). In addition, seroprevalence of 9.4, and 6.9% in buffaloes, and 14 and 11% in humans based on RBPT and ELISA were reported, respectively [17].

False-positive results are the main problem which makes serodiagnosis of brucellosis tedious [18]. A suitable diagnostic test for brucellosis should be inexpensive, fast, sensitive and specific, and labour extensive. For this reason, serological tests are usually applied for the diagnosis of brucellosis [19]. Although, several serological tests have been used for the laboratory testing of brucellosis, no single test is convenient in all epidemiological investigations due to problems of sensitivity (*Se*) and/or specificity (*Sp*) [20, 21]. Rose Bengal Plate test (RBPT) is more sensitive, and often used, but still requires confirmation with other tests [7]. The complement fixation test (CFT) detects IgG antibodies and is used in several countries as a confirmatory test regarding to its higher specificity but may give rise to positive reactions in *B. abortus* S19 vaccinated cattle [22]. Competitive enzyme-linked immunosorbent assay (c-ELISA) has a superior specificity compared to the CFT [23] and had a higher median *Se* (99.0%) and lower *Sp* (95.4%) compared to that of RBPT *Sp* (99.0%) [20] but the assay requires particular equipment and proficient interpretation of results, which may impede its use in many resource-limited countries. Combining c-ELISA and RBPT for the diagnosis of brucellosis is justified because of their relatively high *Se* and *Sp* [20, 21] and the reduction of laboratory and producer costs [24].

The sensitivity (*Se*) and specificity (*Sp*) of serological tests have been found to be influenced by the external environment, such as temperature conditions under which the test is performed, the disease endemic status, animals' vaccination and the presence of cross-reacting antibodies from other Gram-negative bacteria which share similar epitopes with *Brucella* spp. [20, 25, 26].

Studies identifying risk factors for human brucellosis in Pakistan exist [27]. However, possible risk factors in bovines have not been studied yet. This study was conducted to estimate the seroprevalence of bovine brucellosis at the individual animal and herd level, detect *Brucella* DNA in serum by real-time PCR and identify potential risk factors for brucellosis.

## Methods

### Study area and study design

**Fig. 1:**
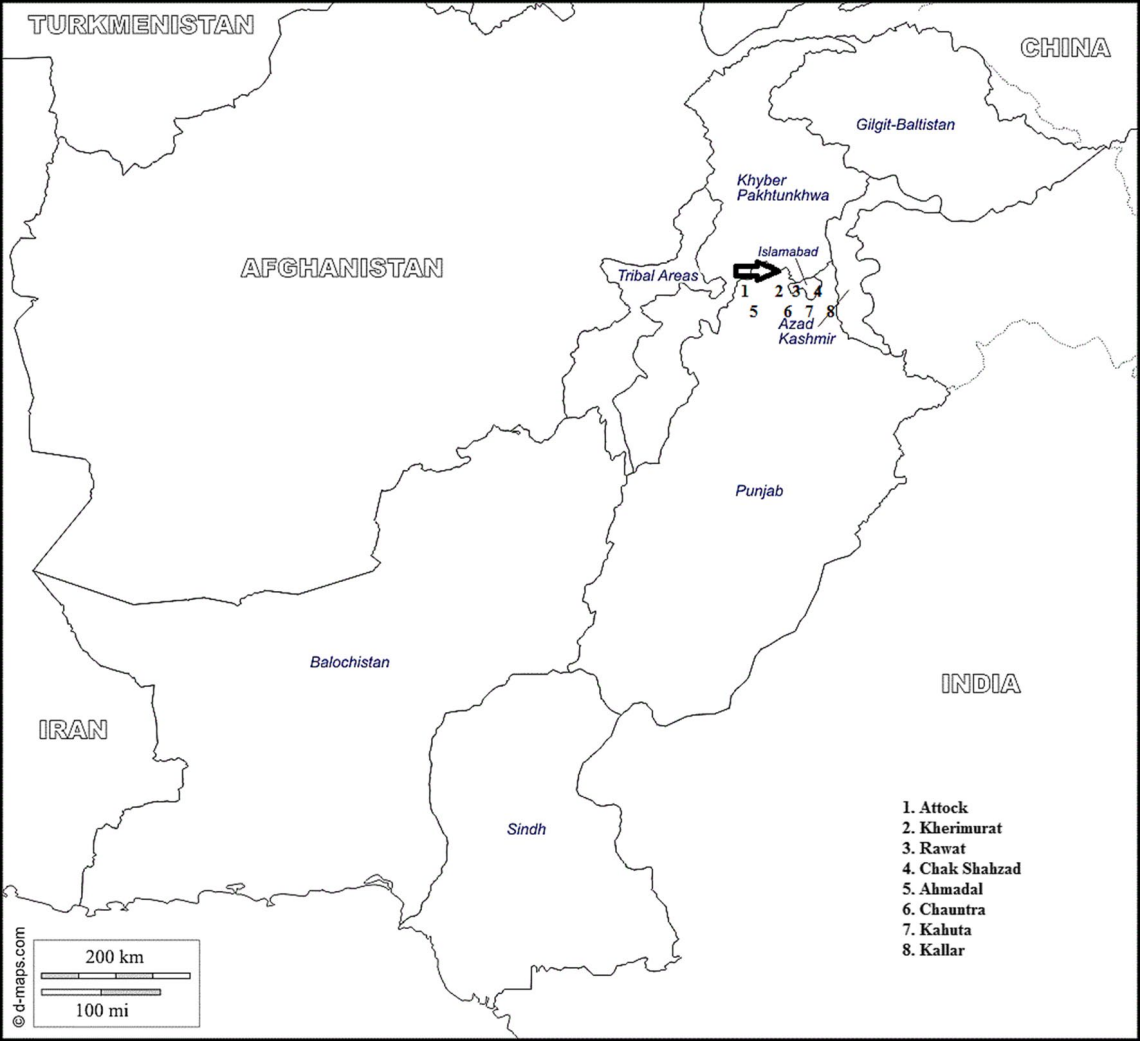
Sampling sites (1–8) from Potoha Plateau, Pakistan. (The map was obtained from http://www.d-maps.com/carte.php?num_car=5567&lang=de)

A cross-sectional study was conducted on the Potohar plateau including Islamabad Capital Territory (ICT), Rawalpindi and Attock districts of Pakistan (Fig. 1). The Potohar plateau is a hilly area having a great diversity of fauna and flora. The area is located in north eastern Pakistan with an elevation of 575 m between the northern part of the Punjab and the western part of Azad Kashmir. Rain water is the main source of irrigation of agricultural land. Other parameters related to the sampling sites have previously been described [28].

This area has all major breeds of buffaloes and cattle of Pakistan which are reared under extensive and semi-extensive grazing systems. According to the 2014–2015 provincial livestock population survey, the number of cattle in this area was estimated to be 19.4 million (49% of total cattle in Pakistan), with 22.5 million buffaloes (65% of total buffaloes in Pakistan), providing more than 67% of the total milk produced in the country [11].

Buffaloes and cattle for blood/milk sampling were selected randomly from eight major sampling locations [Ahmadal (Latitude 33°17′ N; Longitude 72°29′ E), Attock (Latitude 33°46′ N; Longitude 72°21′ E), Chak Shahzad (Latitude 33°39′ N; Longitude 73°8′ E), Chauntra (Latitude 33°30′ N; Longitude 72°22′ E), Kahuta (Latitude 33°34′ N; Longitude 73°22′ E), Kallar (Latitude 33°24′ N; Longitude 73°22′ E), Kherimurat (Latitude 33°30′ N; Longitude 72°52′ E) and Rawat (Latitude 33°29′ N; Longitude 73°11′ E)] located in ICT, Rawalpindi and Attock districts from 2009 to 2011 (Fig. 1). A sample size of 202 herds was calculated expecting a herd seroprevalence of 15.6%, a confidence level of 95% and a desired absolute precision (*d*) of 0.05. Contingencies were taken into account by adding another 25% of animals and herds leading to a total of 253 herds. The 253 herds were randomly selected from the 8 sampling sites due to the lack of a detailed herd and cattle/buffalo identification system. The number of herds was estimated using the formula *n* = (1.96)^2^ *p*(1 − *p*)/*d* ^2^ [29, 30]. The herds were divided into two categories on the basis of the median value of their sizes; below the median value, the herd was considered as “a small holding cluster” (≤8) and above the median value (≥9) as “a large holding cluster”. Herds were of three types, those having only cattle, only buffaloes and those with both cattle and buffaloes (mixed type). Blood/milk samples were collected from 50% of the animals of a herd, for most small holdings, all animals were sampled. To avoid false positives due to the presence of maternal antibodies, only cattle older than 1 year were sampled.

The questionnaire was distributed paper-based through face-to-face interviews (Additional file 1). Data related to age, sex, urbanity, districts/territory, sampling sites, animal species (cattle or buffalo), abortions in third trimester, metritis, herd size, insemination method, source of replacement of animals and body condition of animals were collected at the sampling day. All data were kept for further assessment or if requested.

### Sample collection

A total of 2709 serum samples were randomly collected [1462 buffaloes (53.97%) and 1247 cattle (46.03%)]. Moreover, 2330 milk samples were collected from 1168 cattle and 1162 buffaloes. Approximately 10 ml of blood was collected aseptically from the jugular vein of each animal according to standard procedure [31]. These samples were immediately stored at 4 °C. Samples were then transported to the laboratory. Sera were separated and stored at −20 °C while milk samples were stored at 4 °C.

### Serology

Serum samples were initially screened with RBPT antigens (Institute Pourquier, France). Samples positive to RBPT were confirmed with the serum agglutination test (SAT) (Veterinary Research Institute, Pakistan). All serological tests were performed and results were interpreted according to standard procedures [7, 31, 32]. Briefly, 25 µl of serum were mixed with an equal volume of antigen preparation on a glass plate; the plate was agitated gently for 4 min. A serum sample was considered positive if agglutination occurred. A serum sample positive in RBPT as well as in SAT was considered as positive at the animal level.

SAT was carried out with ethylene diamine tetra acetic acid (EDTA) as described previously [32]. The *Brucella* antigen used in this study was purchased from Immunostics, Inc., USA. One hundred and sixty-eight microliters of Serum Agglutination de Wright (SAW) buffer were added to the first well and 100 μl to the second and third well of a 96-well microtiter plate. 32 µl of test serum was added to the 1st well to reach dilution of 1/6.25. After adequate mixing, 100 μl from the 1st well were transferred to the 2nd well to reach dilution of 1/12.5. Similar to the previous method 100 μl were transferred from the 2nd to the 3rd well to reach dilution of 1/25 and 100 μl discarded from the 3rd well. Then in each well 100 μl of standardized SAW antigen was added giving the serial serum dilutions of 1/12.5, 1/25 and 1/50. The plate contents were thoroughly mixed and incubated for 20–24 h at 37 °C. The value reading was done according to the degree of agglutination [33].

### Milk ring test (MRT)

Milk samples were initially screened by MRT. As per manufacturer recommendations, the MRT antigen was kept at room temperature before use. One milliliter milk sample was added to the test tube. Then 30–40 µL of antigen were added, mixed and incubated at 37 °C for 1 h. A sample having a change in color at the top of the milk was considered positive [7, 31].

### Isolation and identification of *Brucella*

Milk samples considered as positive by MRT were used for isolation of *Brucella*. Isolation was conducted on modified Farrells serum dextrose agar according to standard procedures [31, 34]. Identification and biotyping of these isolates was done according to standard procedures [7, 31, 35].

### DNA extraction and qRT-PCR

Serum samples that tested positive in serology were further subjected to DNA extraction using the High Pure PCR Template preparation kit (Roche Diagnostic, Germany). Purity and concentration of DNA was checked by Nano-Drop ND-1000 UV–Vis spectrophotometer (Nano-Drop technologies, USA). DNA samples were stored at −20 °C until further analysis. A *Brucella* genus-specific (31-kDa salt-extractable immunogenic protein gene, bcsp31) qRT-PCR assay was used for further screening of seropositive samples [36]. Primers and probes were purchased from TIB MOLBIOL (Berlin, Germany). The reactions were conducted in duplicate in microtiter plates (Applied Biosystem, Germany) using M×3000P thermocycler platform (Stratagene, La Jolla, Canada). The thermal profile for assays was 1 cycle of 50 °C for decontamination for 2 min, 1 cycle of 95 °C for initial denaturing for 10 min, 50 cycle of 95 °C for denaturing for 25 s and 1 min for annealing at 57 °C. Cut-off value of cycle threshold (Ct) for a positive sample was ≤40 for *Brucella* genus specific qRT-PCR being automatically generated by the instrument. Herds with at least one animal positive in qRT-PCR were considered as positive.

### Statistical analysis

The true animal (TP) and the herd-level true prevalence of bovine brucellosis were estimated using the Rogan–Gladen formula [37] which uses the apparent prevalence (ratio of the number of seropositive animals to the total number of animals) and accounts for imperfect sensitivity and specificity of RBPT and SAT as:

$$TP = \frac{AP + Sp - 1}{Se + Sp - 1}$$

where *AP* is the apparent animal level or herd level prevalence, respectively, *Se* and *Sp* are the overall animal level or herd level sensitivity and specificity, respectively, based on the serial interpretation of the two tests. At the individual animal level, the overall or combined Se of the two tests based on a serial interpretation is given by

*Se* = *Se*_*RBPT*_ * *Se*_*SAT*_ Whereas the combined specificity is given by *Sp* = *Sp*_*RBPT*_ + (1 − *Sp*_*RBPT*_) * *Sp*_*SAT*_.

To obtain the values for *Se*_*RBPT*_,*Sp*_*RBPT*_, *Se*_*SAT*_ and *Sp*_*SAT*_ to be used in the aforementioned formula, a meta-analytic approach was used. In this approach, a literature search was performed using electronic databases such as Medline, Agricola, CAB international, PubMed and ISI Web of Science. The keywords used in the search included

- RBPT or SAT.
- Diagnostic evaluation.
- Combination of the previous keywords.
- Each combined with bovine brucellosis.

The relevance of selected studies was evaluated using the following inclusion criteria:

- Evaluation of test (s) in question.
- Non-vaccinated cattle populations.
- Sensitivity and specificity estimated.

The data extracted from each selected study included the *Se*, *Sp*, total number of subjects considered from which the number of true positives, true negatives, false positives and false negatives were calculated. These data were then analyzed using “metandi” in Stata 12.1 [38]. The outcome of this analysis is a synthesized estimate (and 95% confidence interval) of the sensitivity and specificity of each test adjusted for the total number of subjects in each of the studies included. The true individual animal level prevalence was estimated across the different potential risk/indicator factors.

Screening of the different potential risk factors related to brucellosis seropositivity was done using univariate random effects logistic regression analysis. Sampling location and herd were both used as random effects to interpret potential clustering of animals within herds and for the differences in herd sizes for the animal level analysis whereas only sampling location was used as a random effect for the herd level analysis to account for clustering of herds within sampling sites. This also accounted for the differences in number of animals within herds and number of herds within sampling regions, respectively. Variables with a *p* value <0.25 in the univariable analysis were further analysed in a multivariable random effects logistic regression model. Manual forward stepwise selection was applied to select the final model using the Akaike’s information criterion (AIC) as the calibrating parameter.

When the removal of a non-significant variable led to a change of more than 25% of the estimated odds ratio, that variable was considered a confounder and was kept in the final model. Multicollinearity was assessed among the independent variables using the Cramer’s phi prime statistic with values >0.7 indicative of co-linearity [39].

All two-way interaction terms of the variables remaining in the final model were assessed for significance based on the likelihood ratio test comparing the model with the desired interaction term and the corresponding model with no interaction terms.

The intra-class correlation coefficient (ICC), which is a measure of the degree of clustering of animals belonging to the same herd or herds belonging to the same sampling location, was computed. In random effects logistic regression models, the individual level variance *δ* ^2^ on the logit scale is usually assumed to be fixed to *π* ^2^/3 [40]. The variability attributed to animals within herds was computed as:

$$ICC_{Herd} = {{{\sigma_{INT:Herd}^{2} }} {\bigg/}{(\sigma_{INT:Herd}^{2} + \pi^{2} /3)}}$$

whereas that attributed to herds within sampling locations was computed as:

$$ICC_{Location} = {{\sigma_{INT:Location}^{2} }}{\bigg/} {{(\sigma_{INT:Location}^{2} + \pi^{2} /3)}}$$

If the ICC is zero, it implies that there is no grouping effect both at the herd and sampling location levels in other words that there is no difference in brucellosis seropositivity among animals within herds and among herds within sampling locations.

The models were built using the xtmelogit function in STATA, version 12.1, software (SataCorp LP, College station, Texas). Model selection was done using Laplacian approximation and the robustness of the final model was assessed by increasing the number of Quadrature (integration) points and monitoring changes in parameter estimates [41, 42].

## Results

### True animal and herd level seroprevalence

The results of the meta-analysis yielded an estimated *Se* and *Sp* of RBT of 0.93 and 0.98, respectively whereas for SAT the estimated *Se* and *Sp* were 0.67 and 0.99, respectively. The overall estimated *Se* and *Sp* based on a serial interpretation of both tests were 0.63 and 1.0, respectively. These values were used to compute the adjusted (true) prevalence.

Out of the 2709 animals, 170 (6.3%) tested positive for *Brucella* antibodies. Seroprevalence at animal level varied from one sampling site to the other i.e. highest in Chak Shahzad region (15.0%) and lowest in Kahuta region (3.9%). Overall 47 (18.6%) herds were found to be positive, among these 19 were cattle herds, 15 were buffalo herds and 13 were mixed herds (Table 1). The corresponding adjusted estimated prevalence was 9.9% (95% confidence interval (CI) 8.4–11.3%). On the other hand, 18.6% (95% CI 14.0–23.9%) of the herds tested were found to be seropositive for brucellosis. When the estimates were adjusted for imperfect test sensitivity and specificity, the corresponding adjusted herd prevalence was 29.3% (95% CI 21.7–36.8%). The cross-classified test results for the number of animals and herds tested, number of positive animals and herds and true prevalence for each of the risk/indicator factors considered are presented in Tables 2 and 3, respectively. Factors associated with animal and herd level seroprevalence on the basis of the univariate random effects logistic regression analysis are presented in Tables 2 and 3, respectively.

**Table 1:** Seroprevalence of brucellosis in individual animals and herds at different sampling sites

Districts/territory	Sampling sites	Animals examined	Animals positive cattle/buffaloes (%)^a^	Herds examined/positive (%)
ICT	Chak Shahzad	260	28/11 (15.0)	10/5 (50.0)
	Rawat	334	10/12 (6.6)	45/6 (13.3)
Rawalpindi	Kallar	399	9/8 (4.3)	43/7 (16.3)
	Chauntra	344	7/12 (5.5)	42/7 (16.7)
	Kahuta	309	7/5 (3.9)	27/3 (11.1)
Attock	Kherimurat	439	7/15 (4.6)	34/8 (23.5)
	Attock	373	11/7 (4.8)	31/7 (22.6)
	Ahmadal	251	8/13 (8.4)	21/4 (19.1)

% Is combined percentage of positive cattle and buffaloes

^a^Number of cattle and buffaloes in sampling site

**Table 2:** Potential risk/indicator factors for animal level brucellosis seropositivity on the basis of univariate analysis

Factors	Levels	Animals examined	Animals positive cattle/buffaloes (total % and 95% CI)	True prevalence (95% CI)	*P value*
Body condition	Healthy	2152	64/63 (5.9: 4.9–7.0)	9.3 (7.7–10.9)	0.443
	Medium	323	15/11 (8.0: 5.3–11.6)	12.7 (8.0–17.4)	
	Weak	234	8/9 (7.3: 4.3–11.4)	11.4 (6.2–16.7)	
Sex	Female	2659	81/77 (5.9: 5.1–6.9)	9.4 (7.9–10.8)	<0.001
	Male	50	6/6 (24.0: 13.1–38.2)	37.8 (19.2–56.5)	
Urbanicity	Rural	1424	46/36 (5.8: 4.6–7.1)	9.1 (7.2–11.0)	0.9624
	Urban	1285	41/47 (6.8: 5.5–8.4)	10.8 (8.6–13.0)	
Age	Young	165	3/4 (4.2: 1.7–8.5)	6.7 (1.8–11.5)	0.129
	Adult	2544	84/79 (6.4: 5.5–7.4)	10.1 (8.6–11.6)	
Animal species	Cattle	1247	87 (6.9: 5.6–8.5)	11.0 (8.8–13.2)	0.036
	Buffaloes	1462	83 (5.7: 4.5–7.0)	8.9 (7.1–10.8)	
Stock replacement	Self-reared	633	27/21 (7.6: 5.6–9.9)	11.9 (8.7–15.2)	0.012
	Purchased	2076	60/62 (5.9: 4.9–7.0)	9.3 (7.7–10.9)	
District/territory	ICT	594	38/23 (10.3: 7.9–13.0)	16.2 (12.3–20.0)	0.164
	Rawalpindi	1052	23/25 (4.6: 3.4–6.0)	7.2 (5.2–9.2)	
	Attock	1063	26/35 (5.7: 4.4–7.3)	9.0 (6.8–11.2)	
Sampling sites	Ahmadal	251	8/13 (8.4: 5.3–12.5)	13.2 (7.7–18.6)	
	Attock	373	11/7 (4.8: 2.9–7.5)	7.6 (4.2–11.0)	
	Chak Shahzad	260	28/11 (15.0: 10.9–19.9)	23.6 (16.8–30.5)	
	Chountra	344	7/12 (5.5: 3.4–8.5)	8.6 (4.9–12.5)	
	Kahuta	309	7/5 (3.9: 2.0–6.7)	6.1 (2.7–9.5)	
	Kallar	399	9/8 (4.3: 2.5–6.7)	6.7 (3.5–9.8)	
	Kherimurat	439	7/15 (5.0: 3.2–7.5)	7.9 (4.7–11.1)	
	Rawat	334	10/12 (6.6: 4.2–9.8)	10.4 (6.2–14.6)

**Table 3:** Potential risk/indicator factors for herd level brucellosis seropositivity based on univariate analysis

Factors	Levels	Herds examined (number positive)	Apparent prevalence (95% CI)	True prevalence (95% CI)	*P value*
Urbanicity	Rural	140 (20)	14.3 (8.9–21.2)	22.5 (13.4–31.7)	0.747
	Urban	113 (27)	23.9 (16.4–32.8)	37.7 (25.2–50.1)	
Presence of animals with metritis	No	241 (42)	17.4 (12.9–22.8)	27.4 (19.9–35.0)	0.057
	Yes	12 (5)	41.7 (15.2–72.3)	65.7 (21.7–100)	
Abortion in third trimester	No	243 (38)	15.6 (11.3–20.8)	24.6 (17.4–31.8)	<0.001
	Yes	10 (9)	90.0 (55.5–99.7)	100	
Herd size	Small	134 (42)	31.3 (23.6–39.9)	49.4 (37.0–61.79)	<0.001
	Large	119 (5)	4.2 (1.4–9.5)	6.6 (0.9–12.3)	
Insemination method	Natural	113 (24)	21.2 (14.1–29.9)	33.5 (21.5–45.3)	<0.001
	Artificial	101 (3)	3.0 (0.6–8.4)	4.6 (0.0–9.9)	
	Both	39 (20)	51.3 (34.8–67.6)	80.8 (56.1–100)	
Districts/territory	ICT	55 (11)	20.0 (10.4–33.0)	31.5 (14.9–48.2)	0.440
	Rawalpindi	112 (17)	15.2 (9.1–23.2)	23.9 (13.4–34.4)	
	Attock	86 (19)	22.1 (13.9–32.3)	34.8 (21.0–48.7)	
Sampling sites	Ahmadal	21 (4)	19.1 (5.4–41.9)	30.0 (3.5–56.5)	
	Attock	31 (7)	22.6 (9.6–41.1)	35.6 (12.4–58.8)	
	Chak Shahzad	10 (5)	50.0 (18.7–81.3)	78.8 (30.0–100)	
	Chountra	42 (7)	16.7 (7.0–31.4)	26.3 (8.5–44.0)	
	Kahuta	27 (3)	11.1 (2.4–29.2)	17.5 (0–36.2)	
	Kallar	43 (7)	16.3 (6.8–30.7)	25.7 (8.3–43.1)	
	Kherimurat	34 (8)	23.5 (10.7–41.2)	37.1 (14.6–59.6)	
	Rawat	45 (6)	13.3 (5.1–26.8)	21.0 (5.4–36.7)	

Out of 170 serological positive samples, 89 (52.4%) were positive using the *Brucella* genus specific qRT-PCR.

Moreover, a total of five isolates were recovered from 156 (6.7%) milk samples of positive animals and these isolates were identified as *B. abortus* biovar 1 according to standard biotyping procedures.

### Risk factors associated with animal and herd level prevalence

The results of the univariable analysis which was based on a random effects model correcting for animal and herd-level clustering indicated that at the individual animal level, sex (cows versus bulls), animal species (cattle or buffaloes) and stock replacement (self-reared versus purchased) were significantly associated with seropositivity at the animal level (*p* < 0.05) (Table 2). At the herd level, herd size and insemination method were significantly associated with seropositivity at the herd level (*p* < 0.05) (Table 2). In addition, the animal level factors i.e. district and age and the herd level factors i.e. presence of animals with metritis were not significant at the 5% level but since their *p* values were <0.25 (Tables 2, 3), they were considered as potential risk factors and thus subjected to the multivariable random effects logistic regression analysis.

The final model for animal level seropositivity included sex, age and stock replacement (Table 4) whereas that for herd level seropositivity included insemination method and herd size (Table 5). The Cramer phi prime estimates indicated no important correlations between any pairs of the independent variables. None of the pair-wise interactions were statistically significant and there were no confounding variables.

**Table 4:** Final model with associated risk factors for brucellosis seropositivity at the animal level-multivariate random effects logistic regression analysis

Factors	OR	(95% CI)	*P value*
*Age*			
Adult versus young	2.4	(1.1–5.5)	0.038
*Sex*			
Females versus males	5.6	(2.6–12.0)	<0.001
*Stock replacement*			
Self-reared versus purchased	1.6	(1.0–2.4)	0.030
	Estimate	95% CI	
*Variance components*			
Herd	0.63	(0.31–1.26)	
Sampling site	8.95e–18

**Table 5:** Final model with associated risk factors for herd level brucellosis seropositivity multivariable random effects logistic analysis

Factors	OR	(95% CI)	*P value*
*Abortion in third trimester*			
Yes versus no	17.4	(1.4–214.1)	0.026
*Insemination method*			
AI versus natural	0.2	(0.1–0.8)	0.027
Both versus natural	4.7	(1.9–11.8)	0.001
*Herd size*			
Large versus small	5.0	(1.74–14.6)	0.003
	Estimate		
*Variance components*			
Sampling site	2.01e–19		

*AI* artificial insemination

Based on the final animal level model, the odds of brucellosis seropositivity were found to be 2.4 (95% CI 1.1–5.5%) times higher among older animals compared to those of the younger animals. In addition, the odds of brucellosis seropositivity were 5.6 (95% CI 2.6–12.0%) times higher among bulls compared to cows. Lastly, animals that were self-reared were 1.6 (95% CI 1.0–2.4%) times more likely to be seropositive for brucellosis compared to those that were purchased. At the herd level, the odds of brucellosis seropositivity were found to be higher for large herds, OR = 5.0 (95% CI 1.7–14.6%) compared to small herds and for herds with occurrence of abortion in third trimester, OR = 17.4 (95% CI 1.4–214.1%) compared to herds with no occurrence of abortion in third trimester, respectively (Table 5). In addition, for herds in which both, AI and natural insemination methods applied, the odds of brucellosis seropositivity were 4.7 times higher compared to those of herds in which natural insemination was practiced. Moreover, the use of AI was found to be a protective factor against brucellosis seropositivity since herds which applied AI were less likely to be brucellosis seropositive, OR = 0.2 (95% CI 0.1–0.8%) compared to those in which only natural insemination was applied.

The value of *σ*_*INT*:*Herd*_^2^, which is the proportion of the total variance in the model explained by the variance between herds, was 0.63 corresponding to an ICC of 0.16 and thus indicating that 16% of the variability in bovine brucellosis seroprevalence occurrs between herds whereas the rest is due to differences between animals within herds. The ICC for sampling location was close to zero indicating that there were no differences in seropositivity of herds based on their sampling sites.

## Discussion

The present study was conducted to investigate the seroprevalence of brucellosis in cattle and buffaloes of different regions of the Potohar plateau, Pakistan at the animal and herd level using serological and molecular methods. Brucellosis vaccination is not practiced in these herds. The seroprevalence of brucellosis at animal and herd level was found to be 6.3 and 18.6%, respectively. These results could be compared with other countries where Brucellosis is prevalent in cattle. For example, in a recent study in Uganda, a lower seroprevalence at animal level (5.0%) and at herd level (6.5%) was found, study that indicated that local herd management is an important factor for the spread of brucellosis [43].

It has to be stressed that the SAT shows lower sensitivity and specificity compared to other standard tests and was not found to be suitable for applying EU legislation intra-Community trade [44]. In developing countries, however, with shortage of diagnostic facilities and limited resources for diagnostic means the serum agglutination test is a cheap and fast tool and was therefore implemented in Pakistani laboratories as well. To increase reliability SAT is used in combination with RBPT.

Among 2709 tested animals, a slightly higher seroprevalence of brucellosis was found in cattle (7.0%) compared to buffaloes (5.7%). A higher seroprevalence was previously reported for cattle (10.18%) and buffaloes (9.38%) using RBPT as screening test in the Faisalabad region of Pakistan [16]. Similarly, a slightly higher seroprevalence was reported in cattle (5.44%) as compared to buffaloes (4.11%) in Egypt [45]. In another study, a high seroprevalence of 14% was found in buffaloes but only 12% in cattle in Egypt [46]. Similar findings related to bovine brucellosis were reported from other countries, i.e., Ethiopia, Zimbabwe and Jordan [9, 19, 47]. Hence, it is not astonishing that variations are seen in seroprevalence at different sampling sites in this study. The overall seroprevalence was found to be higher in animals of the Chak Shahzad area. In this area the husbandry system differed to that of the other sampling sites of ICT, where most of the animals are kept for dairy purposes to cater for the needs of residents of Islamabad and Rawalpindi. Non-lactating animals are immediately replaced with lactating animals to guarantee the milk supply for this region. Non-lactating animals are sent back to their native villages which are several kilometres away from the urban dairy farms. This high turnover caused by frequent replacement might be the cause of the high seroprevalence of brucellosis in this area. In this study animals reared in urban areas were more likely to become seropositive compared to those in rural areas. Higher herd prevalence was also found in the economic zone of Kampala, Uganda (7.4%) when compared to peri-urban (4.1%) and rural areas (6.8%) respectively [43].

Self-reared animals were found to have higher odds of seropositivity in comparison to animals which were purchased from other farms and animal markets. This finding is in contrast with findings from Zimbabwe [19]. In our study, adult animals were found to be more often seropositive when compared to young ones. Similar findings have been reported from other regions of the world where prevalence was higher in mature animals [47]. This finding might be attributed to the increase in exposure with time.

At animal level, age, sex and stock replacement were regarded as potential risk factors in the present study. However, in previous studies, age of animals was found as a risk factor but sex was not confirmed as a risk factor at the animal level [20, 48].

One of the major symptoms of brucellosis in breeding animals in a herd is abortion at an advanced stage of pregnancy (third trimester). In this study herds having animals with a history of abortion especially in the third trimester were found to be associated with higher odds of being seropositive. Similar results were also found in Uganda and Kenya [35, 49]. A high abortion rate and reproductive disorders like metritis were also reported from seropositive herds in Zimbabwe [17, 19, 50].

In the present study, herd size and insemination method were identified as potential risk factors at herd level. Larger herd sizes have been found to be associated with increased odds of seropositivity in urban and peri-urban areas in Uganda and six geographical regions of Zimbabwe [19, 43]. Interestingly, at herd level natural insemination has not been previously reported as a risk factor but in the present study those herds which practiced both (natural and artificial) insemination methods were found to be more often seropositive [20]. Furthermore, in Uganda there was no significant difference found in prevalence in those animals in a herd served with bulls or with artificial insemination [43]. Apart from economic losses due to abortion, a recent study connected “need for repeat insemination” and “birth of weak calves” with seroprevalence in cattle herds in Brazil [51].

*Brucella* genus specific qRT-PCR confirmed the presence of brucellae in the samples. Moreover, our study proved the presence of *B. abortus* biovar 1 in Pakistani bovines on the basis of culture and biotyping.

Due to the imperfect nature of the diagnostic tests used in this study, and the fact that the risk factor analysis based on the random effects logistic regression model could not adjust for this, the significant risk factors identified in this study should be regarded as proxies for the true factors that influence the true prevalence of brucellosis and for many other management factors that were not included in the questionnaire. Detailed observational studies will therefore be needed to confirm the role of each of the identified risk factors on bovine brucellosis seropositivity.

## Conclusion

Different countries successfully eradicated brucellosis from their livestock but brucellosis is at present a persistent problem in Pakistan. No attempts have been made to control brucellosis in or to eradicate it from livestock in this country yet. It is well known that brucellosis in livestock poses also a severe risk for human health. The seroprevalence in this study was 6.3% and varied across different sampling regions. At herd level, herd size, abortion and insemination methods were considered as potential risk factors for brucellosis. While at animal level, sex, age and stock replacement were associated with *Brucella* seropositivity. Detection of *B. abortus* biovar 1 in cattle and buffalo raw milk highlights the significant danger to public health. Although the MRT and RBPT are first line screening tests for brucellosis in livestock in Pakistan, their lack of specificity is of concern. Therefore, the requirement for other more specific confirmatory tests but still fairly cheap should be considered for the control and eradication of brucellosis from livestock in Pakistan, so the risk to humans can be minimized.

## Additional file

**Additional file 1.** Questionnaire for the potential risk/indicator factors for animal level brucellosis seropositivity.

## Abbreviations

B: *Brucella*
CFT: complement fixation test
EDTA: ethylene diamine tetra acetic acid
ELISA: Enzyme Linked Immuno-sorbent Assay
ICI: Islamabad Capital Territory
IgG: Immunoglobulin G
MRT: Milk Ring Test
Qrt-pcr: quantative real time polymerase chain reaction
rbpt: Rose Bengal Plate test
SAT: serum agglutination test
Se: sensitivity
Sp: specificity

## References

[1] Bhat S, Maqbool S, Shah S, Nisar N, Solanki C, Abbas M, Singh S (2012). Brucellosis: A Review. Int J Livest Res..

[2] Nicoletti P (2010). Brucellosis: past, present and future. Prilozi..

[3] Ali S, Ali Q, Neubauer H, Melzer F, Elschner M, Khan I, Abatih E, Ullah N, Irfan M, Akhter S (2013). Seroprevalence and Risk Factors Associated with Brucellosis as a professional hazard in Pakistan. Foodborne Path Dis.

[4] Nabukenya I, Kaddu-Mulindwa D, Nasinyama G (2013). Survey of *Brucella* infection and malaria among Abattoir workers in Kampala and Mbarara Districts, Uganda. BMC Pub Health.

[5] Liu W, Jing Z, Ou Q, Cui B, He Y, Wu Q (2012). Complete genome sequence of *Brucella melitensis* biovar 3 strain NI, isolated from an aborted bovine fetus. J Bacteriol.

[6] Fretin D, Mori M, Czaplicki G, Quinet C, Maquet B, Godfroid J, Saegerman C (2013). Unexpected *Brucella suis* biovar 2 infection in a dairy cow, Belgium. Emerg Infect Dis.

[7] OIE. Bovine Brucellosis. Manual of diagnostic tests and vaccines for terrestrial animals. World Organisation for Animal Health OIE, Paris; 2009. pp. 12–30.

[8] Rhyan J, Nol P, Quance C, Gertonson A, Belfrage J, Harris L, Straka K, Robbe-Austerman S (2013). Transmission of Brucellosis from Elk to cattle and bison, greater yellowstone area, USA, 2002–2012. Emerg Infect Dis.

[9] Al-Majali A, Talafha A, Ababneh M, Ababneh M (2009). Seroprevalence and risk factors for bovine brucellosis in Jordan. J Vet Sci.

[10] Lindahl E, Sattorov N, Boqvist S, Sattori I, Magnusson U (2014). Seropositivity and risk factors for *Brucella* in dairy cows in urban and peri-urban small-scale farming in Tajikistan. Trop Anim Health Prod.

[11] GPMF. Pakistan Economic Survey 2014–2015, Government of Pakistan Ministry of Finance. http://www.finance.gov.pk/survey/chapters_15/02_Agricultre.pdf. Accessed 24 Jan 2017.

[12] Ali S, Ali Q, Melzer F, Khan I, Akhter S, Neubauer H, Jamal S (2013). Isolation and identification of bovine *Brucella* isolates from Pakistan by biochemical tests and PCR. Trop Anim Health Prod.

[13] Shahzad A, Iahtasham K, Qurban A, Ullah KS, Sharif MZ, Shamim A (2014). Isolation and characterisation of Aeromonas sobria in *Catla catla* (Thaila) affected with hemorrhagic septicemia. Bull Eur Ass Fish Pathol..

[14] Munir R, Farooq U, Fatima Z, Afzal M, Anwar Z, Jahangir M (2011). Sero-prevalence of brucellosis in bovines at farms under different management conditions. Br J Dairy Sci..

[15] Shafee M, Rabbani M, Sheikh A, Ahmad M, Razzaq A (2011). Prevalence of bovine brucellosis in organized dairy farms, using milk ELISA, in Quetta City, Balochistan, Pakistan. Vet Med Int..

[16] Shahid M, Basit A, Khan M (2014). Prevalence of Brucellosis among the Hospital Patients of Peshawar, Khyber Pakhtunkhwa. J Infect Mol Biol.

[17] Hussain I, Arshad M, Mahmood M, Akhtar M (2008). Seroprevalence of Brucellosis in Human, Cattle, and Buffalo Populations in Pakistan. Turk J Vet Anim Sci.

[18] Arabaci F, Oldacay M (2012). Evaluation of serological diagnostic tests for human Brucellosis in an endemic area. J Microbiol Infect Dis.

[19] Matope G, Bhebhe E, Muma J, Oloya J, Madekurozwa R, Lund A, Skjerve E (2011). Seroprevalence of brucellosis and its associated risk factors in cattle from smallholder dairy farms in Zimbabwe. Trop Anim Health Prod.

[20] Matope G, Bhebhe E, Muma JB, Lund A, Skjerve E (2011). Risk factors for *Brucella* spp. infection in smallholder household herds. Epidemiol Infect.

[21] Mert A, Ozaras R, Tabak F, Bilir M, Yilmaz M, Kurt C, Ongoren S, Tanriverdi M, Ozturk R (2003). The sensitivity and specificity of *Brucella* agglutination tests. Diag Microbiol Infect Dis.

[22] Nielsen K (2002). Diagnosis of brucellosis by serology. Vet Microbiol.

[23] McGiven J, Tucker J, Perrett L, Stack J, Brew S, MacMillan A (2003). Validation of FPA and cELISA for the detection of antibodies to *Brucella abortus* in cattle sera and comparison to SAT, CFT, and iELISA. J Immunol Meth.

[24] Gall D, Nielsen K (2004). Serological diagnosis of bovine brucellosis: a review of test performance and cost comparison. Rev Sci Tech Off Int Epiz.

[25] Greiner M, Gardner I (2000). Epidemiologic issues in the validation of veterinary diagnostic tests. Prev Vet Med.

[26] Mainar-Jaime R, Muñoz P, de Miguel M, Grilló M, Marín C, Moriyón I, Blasco J (2005). Specificity dependence between serological tests for diagnosing bovine brucellosis in *Brucella*-free farms showing false positive serological reactions due to *Yersinia enterocolitica* O:9. Canad Vet J.

[27] Gwida M, El-Gohary A, Melzer F, Tomaso H, Rösler U, Wernery U, Wernery R, Elschner M, Khan I, Eickhoff M, Schöner D, Neubauer H (2011). Comparison of diagnostic tests for the detection of *Brucella* spp. in camel sera. BMC Res Notes.

[28] Chaudary F, Khan M, Qayyum M (2007). Prevalence of *Haemonchus contortus* in naturally infected small ruminants grazing in the Potohar area of Pakistan. Pak Vet J.

[29] Humphry R, Cameron A, Gunn G (2004). A practical approach to calculate sample size for herd prevalence surveys. Prev Vet Med.

[30] Wagner B, Salman M (2004). Strategies for two-stage sampling designs for estimating herd-level prevalence. Prev Vet Med.

[31] Alton G (1988). Techniques for the brucellosis laboratory.

[32] Garin B, Trap D, Gaumont R (1985). Assessment of EDTA seroagglutination test for the diagnosis of bovine brucellosis. Vet Rec.

[33] Shey-Njila O, Nya D, Zoli P, Walravens K, Godfroid J, Geerts S (2005). Serological survey of bovine brucellosis in Cameroon. Rev Elev Méd Vét.

[34] Farrell I, Robertson L (1972). A comparison of various selective media, including a new selective medium for the isolation of *Brucellae* from milk. J App Bacteriol.

[35] Magona J, Walubengo J, Galiwango T, Etoori A (2009). Seroprevalence and potential risk of bovine brucellosis in zerograzing and pastoral dairy systems in Uganda. Trop Anim Health Prod.

[36] Probert W, Schrader K, Khuong N, Bystrom S, Real-Time Graves M, Multiplex PCR (2004). Assay for detection of *Brucella* spp., *B. abortus*, and *B. melitensis*. J Clin Microbiol.

[37] Rogan W, Gladen B (1978). Estimating prevalence from the results of a screening test. Am J Epidemiol.

[38] Harbord R, Whiting P (2009). metandi: meta-analysis of diagnostic accuracy using hierarchical logistic regression. Stata J.

[39] Holt H, Eltholth M, Hegazy Y, El-Tras W, Tayel A, Guitian J (2011). *Brucella* spp. infection in large ruminants in an endemic area of Egypt: cross-sectional study investigating seroprevalence, risk factors and livestock owner’s knowledge, attitudes and practices (KAPs). BMC Public Health.

[40] Snijders T, Bosker R (1999). Multilevel analysis: an introduction to basic and advanced multilevel modeling.

[41] Frankena K, Somers J, Schouten W, van Stek J, Metz J, Stassen E, Graat E (2009). The effect of digital lesions and floor type on locomotion score in Dutch dairy cows. Prev Vet Med.

[42] Twisk J (2003). Applied longitudinal data analysis for epidemiology: a practical guide.

[43] Makita K, Fèvre E, Waiswa C, Eisler M, Thrusfield M, Welburn S (2011). Herd prevalence of bovine brucellosis and analysis of risk factors in cattle in urban and peri-urban areas of the Kampala economic zone, Uganda. BMC Vet Res.

[44] EFSA (2006). Scientific opinion on “performance of brucellosis diagnostic methods for bovines, sheep, and goats”. EFSA J..

[45] Samaha H, Mohamed T, Khoudair R, Ashour H (2009). Serodiagnosis of brucellosis in cattle and humans in Egypt. Immunobiol.

[46] Hegazy Y, Moawad A, Osman S, Ridler A, Guitian J (2011). Ruminant Brucellosis in the Kafr El Sheikh Governorate of the Nile Delta, Egypt: prevalence of a Neglected Zoonosis. PLoS Negl Trop Dis.

[47] Megersa B, Biffa D, Abunna F, Regassa A, Godfroid J, Skjerve E (2012). Seroepidemiological study of livestock brucellosis in a pastoral region. Epidemiol Infect.

[48] Bayemi P, Webb E, Nsongka M, Unger H, Njakoi H (2008). Prevalence of *Brucella abortus* antibodies in serum of Holstein cattle in Cameroon. Trop Anim Health Prod.

[49] Muendo E, Mbatha P, Macharia J, Abdoel T, Janszen P, Pastoor R, Smits H (2011). Infection of cattle in Kenya with *Brucella abortus* biovar 3 and *Brucella melitensis* biovar 1 genotypes. Trop Anim Health Prod.

[50] McDermott J, Arimi S (2002). Brucellosis in sub-Saharan Africa: epidemiology, control and impact. Vet Microbiol.

[51] Aguiar D, Cavalcante G, Labruna M, Vasconcellos S, Rodrigues A, Morais Z, Camargo L, Gennari S (2007). Risk factors and seroprevalence of *Brucella* spp. in cattle from western Amazon, Brazil. Arq Inst Biol, Sao Paulo.

