# Supplementary material for: Seroprevalence and risk factors associated with bovine brucellosis in the Potohar Plateau, Pakistan
Source: BMC Res Notes. 2017 Jan 28;10:73. doi: 10.1186/s13104-017-2394-2 (PMC5273848; doi:10.1186/s13104-017-2394-2)
Supplement: Supplementary file 1 — Additional file 1. Questionnaire for the potential risk/indicator factors for animal level brucellosis seropositivity. [file 13104_2017_2394_MOESM1_ESM.doc]

#

#### **Name of investigator : _________________________________________________________________**

#### **Name of respondent : __________________________________________________________________**

#### **Date of investigation : __________________________________________________________________**

####

#### **District/Territory: __________________________ Sampling site:** _______________________**____**

**Location code : ____________________________ Geographical coordination : _______________**

**S1 Questionnaire for the potential risk/indicator factors for animal level brucellosis seropositivity**

**INFORMATIONS OF THE HERD**

| - **Herd size** | | | | | |
| --- | --- | --- | --- | --- | --- |
|  | - Small | - Large | |  |  |
| - **Body condition** | | | | | |
|  | - Healthy | - Medium | | - - Weak |  |
| - **Sex** | | | | | |
|  | - Male | - Female | |  |  |
| - **Urbanicity** | | | | | |
|  | - Rural | - Urban | |  |  |
| - **Age** | | | | | |
|  |  Young |  Adult | |  |  |
| - **Animal species** | | | | | |
|  |  Cattle |  Buffalo | |  |  |
| - **Stock replacement** | | | | | |
|  |  Self-reared |  Purchased | |  |  |
| - **District/Territory** | | | | | |
|  |  ICT |  Rawalpindi | |  Attock |  |
| - **Sampling site** | | | | | |
|  |  Ahmadal |  Attock |  Chountra | |  Chak Shahzad |
|  |  Kahuta |  Kallar |  Rawat | |  Kherimurat |
| - **Animals with metritis** | | | | | |
|  |  No |  Yes |  | |  |
| - **Abortion in third trimester** | | | | | |
|  |  No |  Yes |  | |  |
| - **Insemination method** | | | | | |
|  |  Natural |  Artificial |  Both | |  |
